# Mental State Assessment and Validation Using Personalized Physiological Biometrics

**DOI:** 10.3389/fnhum.2018.00221

**Published:** 2018-06-01

**Authors:** Aashish N. Patel, Michael D. Howard, Shane M. Roach, Aaron P. Jones, Natalie B. Bryant, Charles S. H. Robinson, Vincent P. Clark, Praveen K. Pilly

**Affiliations:** ^1^Center for Human Machine Collaboration, Information and Systems Sciences Laboratory, HRL Laboratories, LLC, Malibu, CA, United States; ^2^Psychology Clinical Neuroscience Center, The University of New Mexico, Albuquerque, NM, United States

**Keywords:** computational models, machine learning, mental fatigue, stress, attention, validation, N-back recall, EEG

## Abstract

Mental state monitoring is a critical component of current and future human-machine interfaces, including semi-autonomous driving and flying, air traffic control, decision aids, training systems, and will soon be integrated into ubiquitous products like cell phones and laptops. Current mental state assessment approaches supply quantitative measures, but their only frame of reference is generic population-level ranges. What is needed are physiological biometrics that are validated in the context of task performance of individuals. Using curated intake experiments, we are able to generate personalized models of three key biometrics as useful indicators of mental state; namely, mental fatigue, stress, and attention. We demonstrate improvements to existing approaches through the introduction of new features. Furthermore, addressing the current limitations in assessing the efficacy of biometrics for individual subjects, we propose and employ a multi-level validation scheme for the biometric models by means of *k*-fold cross-validation for discrete classification and regression testing for continuous prediction. The paper not only provides a unified pipeline for extracting a comprehensive mental state evaluation from a parsimonious set of sensors (only EEG and ECG), but also demonstrates the use of validation techniques in the absence of empirical data. Furthermore, as an example of the application of these models to novel situations, we evaluate the significance of correlations of personalized biometrics to the dynamic fluctuations of accuracy and reaction time on an unrelated threat detection task using a permutation test. Our results provide a path toward integrating biometrics into augmented human-machine interfaces in a judicious way that can help to maximize task performance.

## 1. Introduction

Human physiological monitoring has become ubiquitous in wearable consumer devices, measuring biomarkers such as heart rate, galvanic skin response, and movements (Raskovic et al., 2004). These devices can provide accurate real-time biofeedback and forensic data useful to athletes, students, soldiers, pilots, and others as an aid to improve performance. The primary limitation of current monitoring solutions, however, is an inability to interpret and validate measured physiological biometrics in the context of task performance. While standard age-related guidelines for metrics such as heart rate for cardiovascular fitness are available (Ziegler et al., 1992; Ryan et al., 1994), such generic population-level guidance is not sufficient for advanced interfaces to accurately predict and enhance near-term user performance on mental tasks. For example, pilot associate systems in aircrafts and military decision aids rely critically on precision biometrics that reflect the physiological mental states of an individual user (Horst et al., 1990; Wilson, 2002; Camhi, 2004). The Air Force Research Laboratory (AFRL) introduced the Sense-Assess-Augment taxonomy to organize their research portfolio around sensing human performers and assessing when a system should intervene to augment their performance (Galster and Johnson, 2013). Such real-time human-machine interfaces can be improved by better mental state predictions. The prediction capabilities described here can inform behavioral interventions, enable training systems that are more engaging and effective for students, automate support systems that can assume control when a driver or pilot is in stress, or more effectively prescribe breaks in taxing environments such as those experienced by air traffic controllers.

Many techniques for assessing and predicting mental states have been proposed (Gevins and Smith, 2000; Healey and Picard, 2005; Oken et al., 2006; Braboszcz and Delorme, 2011; Cheng and Hsu, 2011; Putman et al., 2014; Wascher et al., 2014; Trejo et al., 2015; Ki et al., 2016). Current challenges for existing approaches include not only the lack of a unified method to assess various mental states but also the lack of a procedure to validate the efficacy of their biometric predictions for use in real life. Based on prior literature, we have identified a small set of mental states—attention, mental fatigue, stress—that have a significant effect on behavioral performance. We evaluated and sought to improve upon the current state-of-the-art features for these mental states to build personalized models that can predict task performance on novel generic tasks using data from a minimal set of biosensors: electroencephalography (EEG) and electrocardiography (ECG). We will describe the various biometric models that were considered and a set of validation tests that allow for a standardized comparison.

Attention is a measure of the ability to filter out distractions and focus on task-related items. It has been described as an ability to achieve certainty in perception (Feldman and Friston, 2010). A group at NASA Langley (Pope et al., 1995; Prinzel et al., 2000) developed a metric called “engagement index” based on a proportion of EEG power bands *beta*/(*theta*+*alpha*) to adaptively control the allocation of tasks in an adjustable autonomy setting. Braboszcz and Delorme (2011) established two attentional states: a high vigilance state and a low-alertness mind-wandering state, with the latter characterized by increased EEG spectral power in the *theta* and *delta* bands and decreased power in the *alpha* and *beta* bands. The subjects were instructed to count breaths and to press a key whenever they realized they were mind-wandering. EEG data from 8 to 2 s before each mind-wandering event is labeled as mind-wandering, and the data from 2 to 8 s after each event is labeled as attentive. This breath-count task is adopted for the present study. Putman et al. (2014) defined attention as a top-down ability to overcome bottom-up distractors for effective task performance and proposed that an effective biomarker for attentional control is a proportion of spectral power in the *theta* and *beta* bands, averaging EEG data from three frontal electrodes. Ki et al. (2016) found that EEG *alpha* band (10 Hz) power is modulated by attention independent of task or stimulus. Oken et al. (2006) reviewed prior literature and focused on vigilance as a state of sustained attention or tonic alertness. They noted that increased *theta* and decreased *beta* power in EEG most often correlate with worse performance on a sustained attention task, which agrees with the findings of Braboszcz and Delorme (2011) and Putman et al. (2014). None of these studies developed a model to classify and validate the attention level on other novel tasks; but based on these studies, we chose attention as one of our key biometrics, and evaluated the *theta*/*beta* proportion in one of the models.

Mental fatigue has a direct relationship to cognitive performance (Kato et al., 2009; Trejo et al., 2015). Trejo et al. (2015) found an association between mental fatigue and increase in the EEG spectral power of frontal *theta* and parietal *alpha* bands. They estimated fatigue using a partial least squares model coupled with a discrete-output linear regression classifier. Wascher et al. (2014) noted a mental fatigue-related increase in frontal EEG *theta* power as well in occipital EEG *alpha* power. *Theta* band power reliably and continuously increased with time on the task through a 4-h experimental period, but *alpha* band power increased faster, peaking after 1.5 h and remaining steady for the rest of the time. Wilson and Russell (2003) used a neural network model on five bands each of EEG and EOG data crossing most of the spectrum in 10 s windows with 50% overlap, but they got the best results with EEG alone. Gevins and Smith (2000, 2003) and Smith et al. (2001) proposed a workload metric (“Task Load Index”, TLI) as the ratio of frontal midline EEG *theta* to parietal *alpha* power.

Stress has been known to be a critical component of mental state; however, a precise definition of stress is elusive. It has been characterized as the body's physiological response to environmental challenges: threats to tangible or intangible resources that are valued by the individual, or depletion of those resources (Hobfoll, 1989). Stress caused by a current task is added on top of background stress that may be caused by life events. In general, the relationship between stress and performance takes the form of an inverted U-curve (Hebb, 1955). Individual responses to stress vary widely but task performance is best under moderate stress (at the center of curve), and either low stress or high stress is detrimental to performance (Roberts et al., 2016). Stress can be detected in a number of physiological responses including galvanic skin response (GSR) (Healey and Picard, 2005), saliva cortisol level, and pupil dilation (Henckens et al., 2009). Healey and Picard (2005) experimented with electromyography (EMG), electrocardiography (ECG), skin conductance, and respiration with varying success. Hockey et al. (2009) and Ting et al. (2010) designed a fuzzy decision system that monitors the TLI metric mentioned above, and heart rate variability (HRV), to assess the “operator functional state” for deciding whether a complex system should be controlled by a human or an automated system to reduce operational risk.

Focusing on attention, mental fatigue, and stress as the most pertinent biometrics affecting cognitive behavior, we have designed a unified pipeline to construct personalized models based on two intake experiments. These models can subsequently be used to produce quantified measures of the three biometrics during task performance. We propose that integrating all available neural and physiological features into a meaningful score for each biometric would allow for the most accurate measures. The intake experiments are breath-count, a variant of the Braboszcz and Delorme (2011) method described above, and *N*-back recall (Kirchner, 1958). Breath-counting is a relaxing task that causes minimal cognitive load, but requires sustained attention. And because breath-counting lowers stress and fatigue, it is used as a baseline low level for training stress and mental fatigue models. The visual-letter *N*-back recall task is used to evoke two different levels of cognitive workload; 1-back is moderately taxing, and 3-back is the most taxing. Data required for the proposed metrics is limited to physiological features extracted from EEG and ECG data; however, the methods proposed allow for scaling up the input features for each component of mental state to include other measurements such as GSR and pupilometry. To address the challenges in physiological assessment, we validated the proposed models using *k*-fold cross-validation and regression testing. After training models on the simple laboratory intake experiments, we demonstrated their use in an unrelated, novel threat detection task, which is a complex real-world perceptual learning task (illustrated in **Figure 2**). The task is based on a virtual reality training system for soldiers called “DARWARS Ambush!” (MacMillan et al., 2005), and has been used in previous studies aimed at enhancing the efficacy of training (Clark et al., 2012; Coffman et al., 2012). This is a relevant task for our purposes because it is fatiguing, requires sustained attention, and a failure to detect a threat results in a stressful confrontation.

## 2. Methods

Data from the breath-count (Braboszcz and Delorme, 2011) and *N*-back recall (Kirchner, 1958) tasks of 18 subjects (Table S1) were used to train models for each of the three metrics: mental fatigue, stress, and attention. All subjects participated in a purely voluntary manner after providing informed written consent under experimental protocols approved by the Chesapeake Institutional Review Board. On the evening of the first day, each subject performed a breath-count task lasting 30 min followed immediately by a 3-back recall test lasting 21 min. The subject then rested in a sleep laboratory for 8 h during the night, and the following morning a 1-back recall task was administered lasting 21 min.

**Figure 1:**
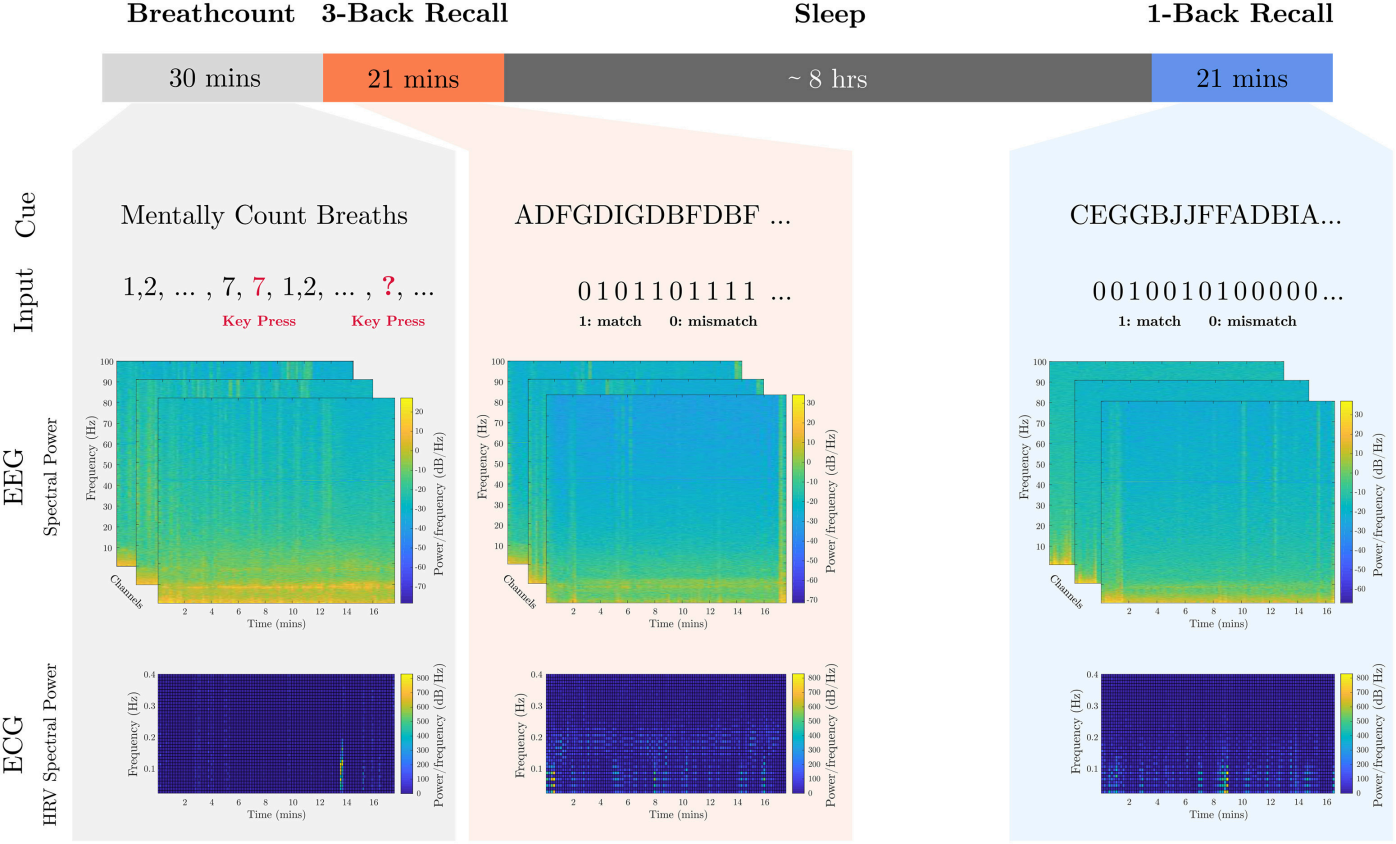
Calibration tasks overview. The timeline of the calibration tasks is shown. An 8-h sleep period where participants were under observation in a sleep laboratory separates the 1-back recall task from the breath-count and 3-back recall tasks. Sample cue and participant inputs are shown below the respective tasks. Sample EEG channel spectrograms aligned to the start of the task are shown, as well as sample HRV spectrogram data extracted from the ECG data.

The breath-count task (Braboszcz and Delorme, 2011) requires sustained attention but minimal cognitive load. As it is similar to mindful breathing exercises used to reduce stress and induce a relaxation response (Lum, 1977; Feldman et al., 2010), it is utilized for training the baseline levels of low mental fatigue and low stress as well. Prior to the session, subjects were told to repeatedly count their breaths mentally from 1 to 10. If they count past 10 or lose count, they were instructed to press a button, refocus, and restart counting breaths. Mental workload was applied by means of the visual-letter *N*-back recall task, which exploits working memory constraints (Kirchner, 1958). Subjects were positioned in front of a screen where written instructions were displayed. The individuals were informed that a series of characters between A and J would be presented randomly, and they were to indicate if the new character matched the one presented *N* steps prior. In separate tasks, a moderate loading was measured with *N* = 1, and a heavy cognitive loading was measured with *N* = 3. During testing, the screen displayed characters one at a time in 12-point Tahoma font in black on a light gray background. Each letter was presented for 500 ms, with an inter-stimulus interval of 1,000 ms. A timeline of the calibration tasks including example collected data is presented in Figure 1.

**Figure 2:**
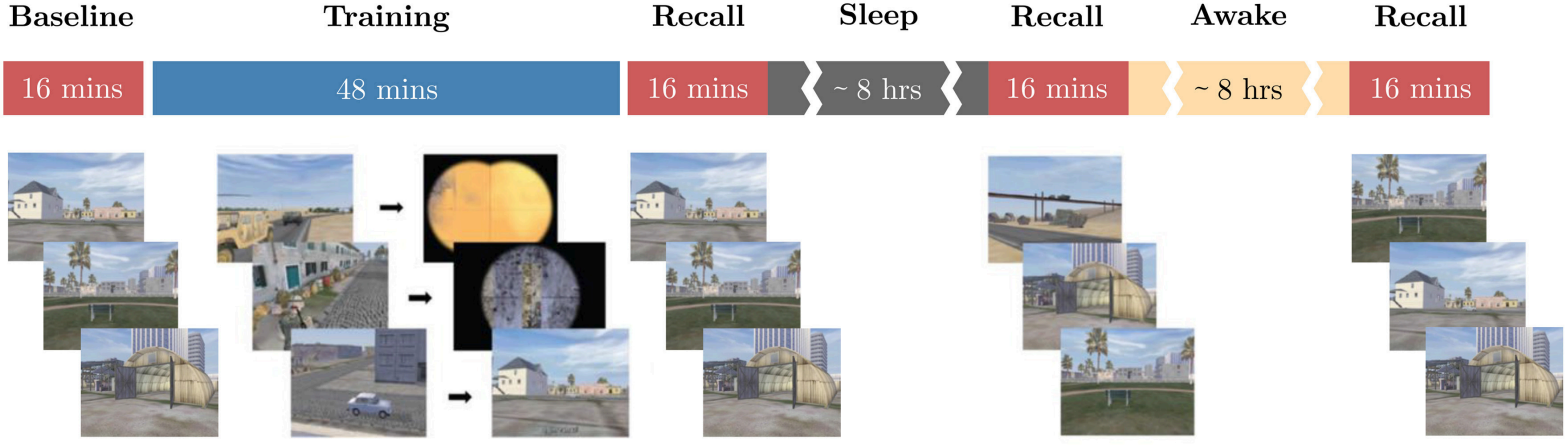
Threat detection task overview. The timeline of the threat detection task used for novel task validation of the biometric models is shown. Red blocks correspond to sessions where no feedback is provided to the subjects and blue blocks to where feedback is provided. While sessions are back-to-back, there are short breaks between them where the experimenter ensures that the subjects understand the instructions. The content below each block depicts sample images shown to the subjects during the respective block. Note the timeline for only one experimental day of the within-subjects design is shown here.

To demonstrate how these biometric models are validated using data from a novel task, we presented a threat detection task (Jones et al., under review) to the subjects on two subsequent 24-h periods separated by 3–7 days. The task required participants to identify the presence of threat cues in still images taken from the “DARWARS Ambush!” virtual reality training environment (MacMillan et al., 2005), with a yes or no response for each image presented for 2 s. One of two types of threats was shown on each experimental day: people threats including enemy combatants, snipers, etc., vs. object threats including IEDs, other explosive devices, trip wires, laser sightings, etc. On each day, participants were first assessed using test images without feedback to obtain their baseline level of knowledge regarding the threat cues from the given threat type. They were then given 45 min of training, during which they not only viewed each image and made a response, but also received feedback in the form of a short movie. If a threat was present and detected, or if no threat was present and the correct response was given, the subject was shown a movie with a positive outcome and a voice-over informing participant that they were doing well. If a threat was present and not detected, the movie showed the deadly consequences and the voice-over berated the subject. Depending on the threat for that trial, feedback might include an IED exploding, a sniper shooting, a soldier falling, etc. Finally, if a threat was not present and the wrong response was given, the participant was shown a video with no bad outcome, but with a slightly negative voice-over feedback. After training, subjects were tested again in three sessions without feedback: immediately after training, in the morning after sleep, and once in the afternoon (about 24 h after training). Figure 2 shows the task timeline and sample scenario images. Number of trials for each threat type that were available for evaluation of the biometric models on the threat detection task can be found in Table S2, and a complete task description can be found in Jones et al., (under review).

### 2.1. Biometric assessment

EEG and ECG were collected from all participants using a 32-channel Neuroelectrics StarStim32 system sampled at 500 Hz. The 32 Ag-AgCl electrodes were arranged in the international 10–20 system (P7, T7, CP5, FC5, F7, F3, C3, P3, FC1, CP1, Pz, PO4, O2, Oz, O1, PO3, CP2, Cz, FC2, Fz, AF3, Fp1, Fp2, AF4, P8, T8, CP6, FC6, F8, F4, C4, P4) and held in place with a neoprene EEG cap. Three of the 32 were utilized as external channels; namely, ECG (PO3) placed under the left collarbone, and both vertical (AF3) and horizontal (AF4) electro-oculogram (EOG): one placed superior and lateral to the right outer canthus, and another inferior and lateral to the left outer canthus. Two reference electrodes (CMS and DRL) were placed preauricularly. Pre-processing of EEG data was performed with an automated artifact removal pipeline (Ketz et al., 2017). As part of this, EEGLAB (Delorme and Makeig, 2004) was utilized to perform automatic channel rejection based on outliers in normalized spectral power within the 1–250 Hz range (> mean + 3 standard deviation), along with band-stop filtering from 59 to 61 Hz, high pass filtering above 0.1 Hz, DC offset removal, and average re-referencing. Next, again utilizing EEGLAB (Delorme and Makeig, 2004), Independent Component Analysis (ICA) was performed following which the Independent Components (ICs) were ranked as candidate noise components by their correlation with nuisance signals (accelerometer, EOG, ECG) using the SemiAutomatic Selection of Independent Components for Artifact correction (SASICA) plugin for EEGLAB (Chaumon et al., 2015). At several stages of the process, EEG quality was assessed using the Signal Quality Index (SQI) (Daly et al., 2012, 2013). Noise ICs were iteratively selected where the top ranked component was first added to the noise components list and subtracted from the pre-processed data. If the change in SQI following the removal was less than −0.001 (a quality increase), that IC remained on the noise components list; if not, it was removed. The next highest ranking IC was then added to the noise components list, and the process proceeded in this fashion until all the ICs had at one time been a part of the noise components list, or there were a minimum 25% of the ICs not selected as noise components. The remaining, non-noisy components were then back-projected to the channel space to recover the topological signal. Lastly, rejected channels were replaced by spherical interpolation of nearby channel data followed by moving average subtraction with a window of 1000 samples for EEG and 50 samples for ECG (for drift correction and to mean center the signals at 0). Finally, a Fast Fourier Transform was applied to the time series data to extract spectral power in different frequency bands (see Table 1) from 10 s non-overlapping bins for training the mental fatigue and stress models, and from 6 s windows before and after self-reports of mind-wandering in the breath-count task (see **Figure 5**) for training the attention models. Classifiers were then trained on these data for each electrode separately to compute the various biometrics as described below. The final classifications were computed by taking the mean value of the output predictions across all the electrodes, except for models based on ECG alone (namely, the first two models for stress: $P_{s}^{1}$ and $P_{s}^{2}$). The testing of models was performed on 10 s overlapping segments at 1 s intervals for mental fatigue and stress, and on 6 s overlapping segments at 1 s intervals for attention.

**Table 1:** EEG frequency ranges for informative spectral bands.

**Band**	**Frequency range (Hz)**
δ (delta)	1–3
θ (theta)	4–8
α (alpha)	8–12
β (beta)	13–30
γ (gamma)	30–50
Γ (high-gamma)	50–100

In the following discussion, our notational convention for biometrics will be $P_{b}^{n}$, where *b* is either attention (*a*), mental fatigue (*f*), or stress (*s*), and *n* is a numeric designation for each variant of the biometric model. The input data for each of the biometric models is described in the next 3 subsections, and the section 2.2 discusses the classifiers trained on each of the feature sets and the validation techniques used to evaluate the effectiveness of the different models.

#### 2.1.1. Mental fatigue

**Figure 3:**
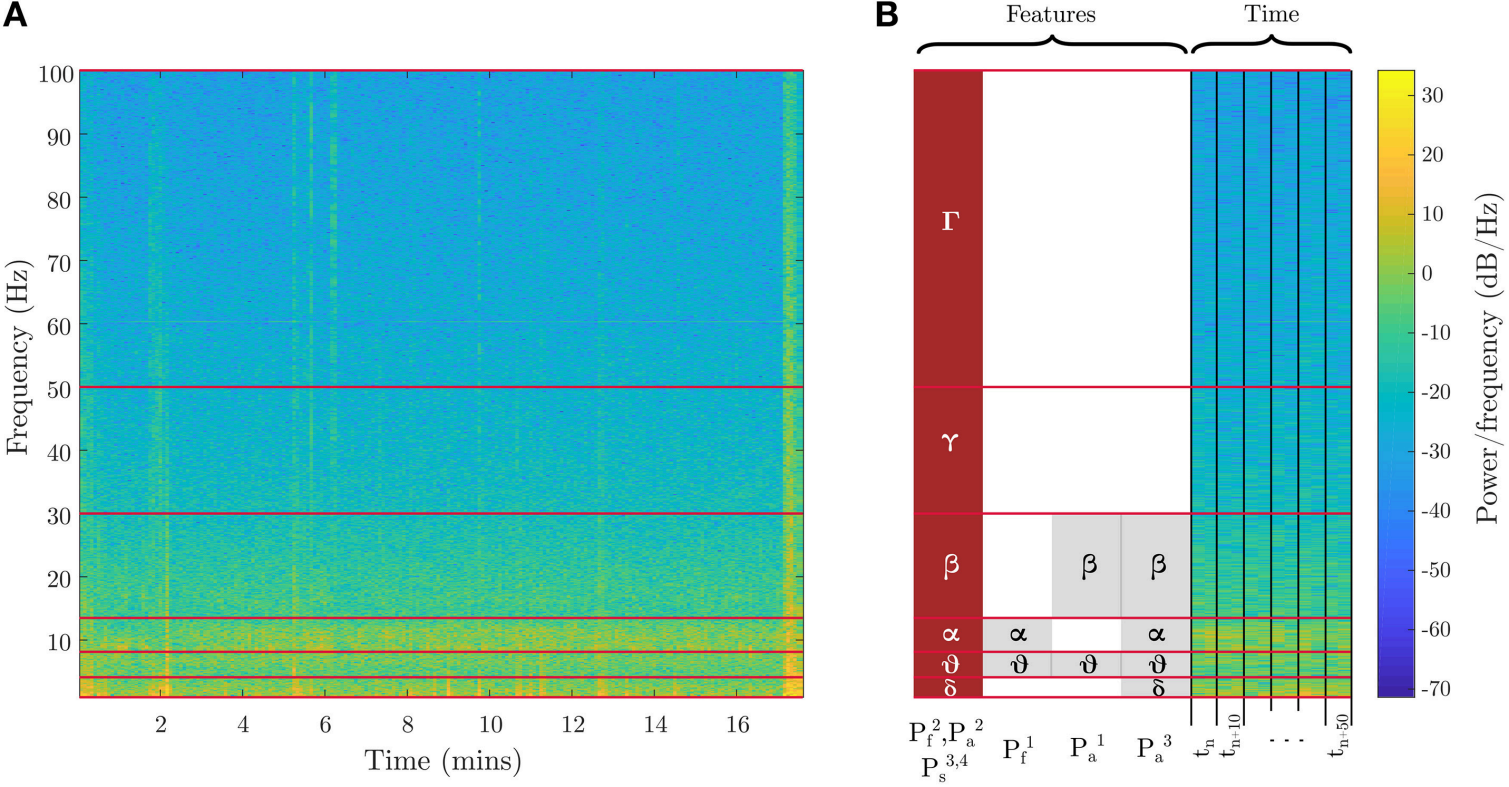
EEG features for fatigue, attention, and stress metrics. Spectral power features in different frequency bands used in two variants of the fatigue metric, three variants of the attention metric, and one variant of the stress metric are illustrated. **(A)** shows the spectrogram from the full evaluation period for a sample channel on the 3-back task. **(B)** shows a zoomed-in segment outlining the time segmentation and feature binning for the different biometric models. The red blocks are the best EEG features, and the gray blocks are other evaluated features. Table 1 defines the frequency ranges.

The mental fatigue (*P*_*f*_) induced by cognitive loading rises steadily over time. The breath-count task provides the representative relaxed baseline state and 3-back task produces fatigue. To ensure sufficient loading from the 3-back recall task, only the latter half of the recorded data is utilized. For the mental fatigue models, we utilized spectral power in different frequency bands as features with discrete labels, {0,1}, to identify low fatigue (from the breath-count task) vs. high fatigue (from second half of 3-back). The features for the two evaluated fatigue models are shown in Figure 3 and defined as follows (frequency bands are shown in Table 1):

- $P_{f}^{1}$*:* Average EEG spectral power in *theta* and *alpha* bands, per Trejo et al. (2015)
- $P_{f}^{2}$*:* Average EEG spectral power across all bands from *delta* through *high-gamma*

#### 2.1.2. Stress

**Figure 4:**
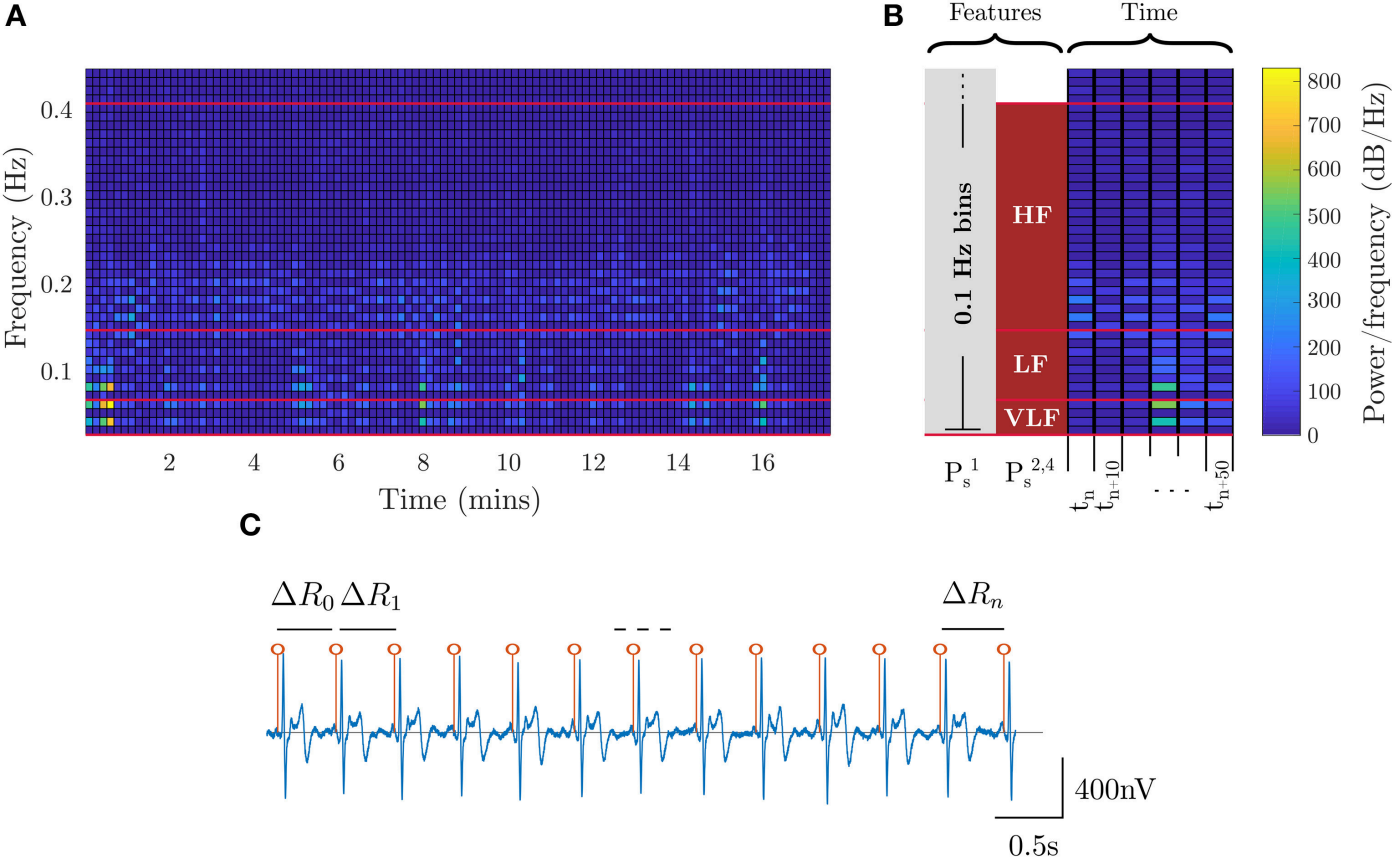
ECG features for stress. **(A)** shows the HRV spectral features extracted from ECG. The red lines, and corresponding blocks, identify the best features for assessing stress. **(B)** shows a zoomed-in segment outlining the time segmentation and feature binning for the different stress models. **(C)** shows the R-R peak detection for the ECG waveform where the interval between peaks is utilized as the HRV signal.

The stress metric (*P*_*s*_) returns a low value on relaxing tasks and high on challenging tasks. Accordingly, models for stress are learned from the low-stress breath-count task and the high-stress 3-back task. In addition to the EEG data, ECG data is also employed to assess the physiological response to stressors. Heart rate variability (HRV) is extracted from ECG (Healey and Picard, 2005) by detecting the QRS waveforms and computing the interval between consecutive beats (Figure 4). For the stress models, we utilized specific bands in the HRV power spectrum that have an underlying interpretation; namely, lower-frequency (*f*_*HRV*_ ≤ 0.08 Hz) spectral power indicates the influence of the sympathetic nervous system, and higher-frequency (0.15 Hz ≤ *f*_*HRV*_ ≤ 0.5 Hz) power indicates the influence of the parasympathetic nervous system (Healey and Picard, 2005). Additional features were drawn from EEG spectral power in different frequency bands. The ECG and neural features are visualized in Figure 4 and in Figure 3, respectively.

The features for the four evaluated stress models are defined as follows:

- $P_{s}^{1}$*:* Spectral power of ECG frequencies with 0.1 Hz binning (motivated by Liu et al., 2006) in the 0.01–0.4 Hz range
- $P_{s}^{2}$*:* Average HRV spectral power in VLF, LF, and HF bands (Table 2, per Healey and Picard, 2005)
- $P_{s}^{3}$*:* Average EEG spectral power across all bands from *delta* through *high-gamma* (Table 1)
- $P_{s}^{4}$*:* $P_{s}^{2}+P_{s}^{3}$

**Table 2:** HRV frequency ranges for informative spectral bands.

**Band**	**Frequency range (Hz)**
VLF (Very Low Frequency)	0.01–0.04
LF (Low Frequency)	0.04–0.15
HF (High Frequency)	0.15–0.4

#### 2.1.3. Attention

**Figure 5:**
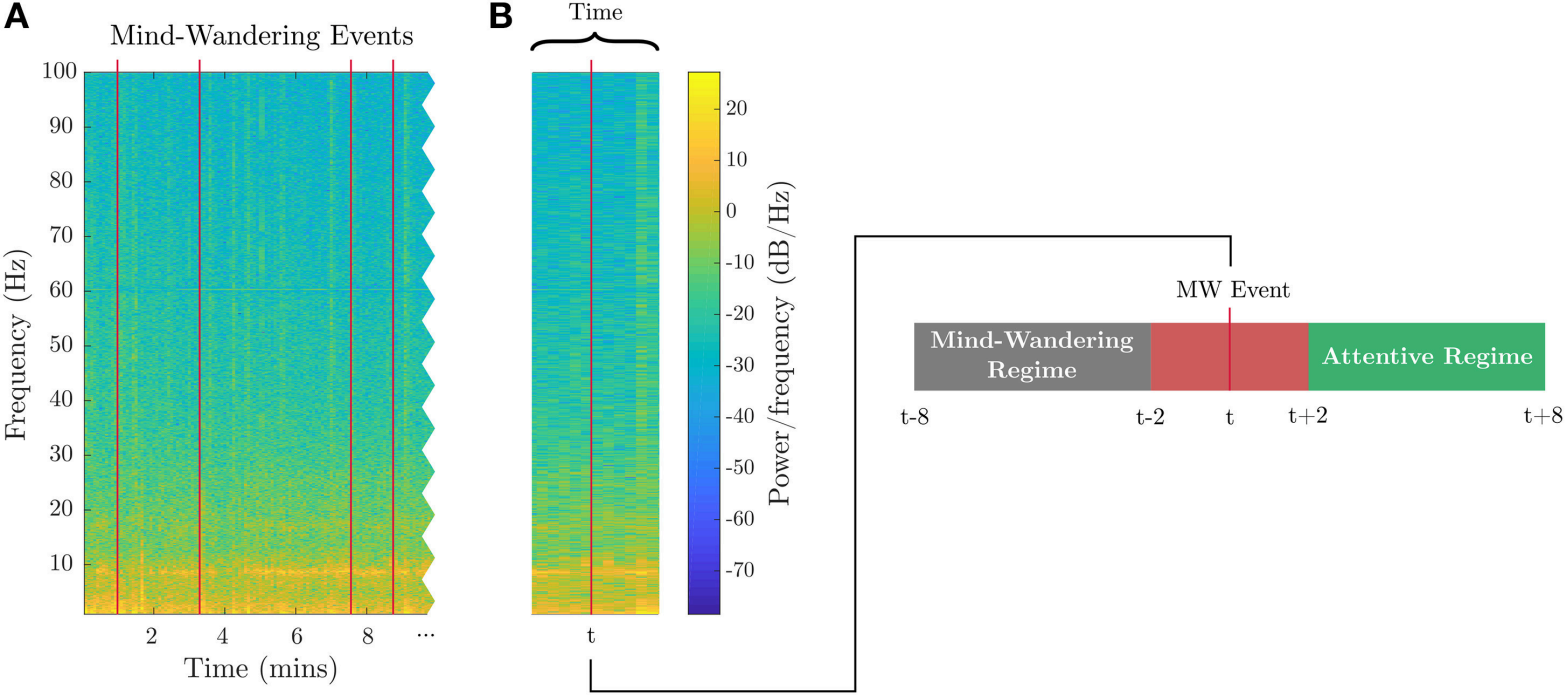
EEG features for attention. **(A)** Subject-identified mind-wandering events are plotted as red lines in the spectrogram of EEG data from a sample channel during the breath-count task. **(B)** Example of one of the mind-wandering events at time t, with relative windows labeled as inattentive (mind-wandering) prior to user button press and attentive occurring after the button press.

The attention metric, *P*_*a*_, is computed exclusively from the breath-count task. Each mind-wandering event indicated by the subject is treated as an event related potential marker. Nine subjects of 18 were excluded for having less than three mind-wandering events. As in Braboszcz and Delorme (2011), neural data taken from 8 to 2 s before the button press are labeled as inattentive, and data from 2 to 8 s after each button press are labeled as attentive. Figure 5 shows marked data with the timeline for a sample mind-wandering event. If the time windows for inattention and attention were ever to overlap, we would consider the windows as data for inattention because the button press did not elicit an expected bout of sustained attention. For the attention models, we utilized average EEG spectral power in various frequency bands as features, including the *theta*/*beta* proportion ($P_{a}^{1}$) proposed by Putman et al. (2014), and with discrete labels, {0,1}, to identify inattentive vs. attentive states. The features for the three evaluated attention models are defined as follows (frequency bands are shown in Table 1):

- $P_{a}^{1}$*: theta*/*beta* proportion, per Putman et al. (2014)
- $P_{a}^{2}$*:* Average EEG spectral power across all bands from *delta* through *high-gamma*, per Wilson and Russell (2003), although they also included EOG
- $P_{a}^{3}$*:* Average EEG spectral power in *delta, theta, alpha*, and *beta* bands, per Braboszcz and Delorme (2011).

### 2.2. Modeling

The neural and cardiac data used to train the models have a great amount of variability across subjects due to the differences in the underlying physiology and health of the individuals. Different statistical models were considered to evaluate the different feature sets for each mental state component defined above. For situations where discrete classification is a sufficient characterization, the following models were evaluated with categorical outputs: linear discriminant analysis (Duda et al., 2001), and support vector machine with linear and radial basis kernels (Chang et al., 2010). Where discrete classification is not a sufficient output, regression was considered with the following models evaluated: linear regression, epsilon-support vector regression (Basak et al., 2007), and generalized linear model (GLM, McCullagh and Nelder, 1989) using a binomial distribution and a logit link function. Of the considered models, GLM had the best performance for both discrete and continuous classification and is the model of choice for the presented results.

To evaluate the discrete classification models, standard *k*-fold cross-validation was used to assess the classification accuracy of each feature set for the three mental state components. Data was separated into 10 partitions and permuted exhaustively with 9 partitions used for training and one partition for testing. Each partition contained an equal number of samples per class, and a uniform prior was utilized. Each channel was evaluated independently, and results were averaged across channels per subject. To evaluate the regression models, each model was trained on the same data utilized for the best feature sets determined from the *k*-fold validation process for mental fatigue and stress. Leave-one-out cross-validation provided bounds for each biometric. Data from the 1-back recall task, which was not utilized for training, served to evaluate whether a continuous mapping was learned for the given biometric. It is expected that if the biometric models are valid, the 1-back recall data should be rated at an intermediate value somewhere between the relaxing breath-count task and the more stressful and fatiguing 3-back recall task. Five subjects of 18 were excluded in the regression validation for either a lack of neural recordings (one subject) or excessively noisy ECG recordings despite artifact removal (four subjects) for the 1-back recall task.

#### 2.2.1. Evaluation on novel threat detection task

The best biometric models from the *k*-fold cross-validation were applied to a novel task to test if the biometrics correlate with dynamic fluctuations in performance. We measured the correlation between the temporal dynamics of each predicted biometric and the subject's performance (accuracy and reaction time) on the threat detection task described in Figure 2. Four subjects of 18 were excluded from analysis for excessively noisy EEG or ECG recordings despite artifact removal. The task data was segmented to match the threat detection trials, and average biometric values were computed for each segment. The data for the two threat types, namely object and person threats, were collected on separate days per subject and are evaluated independently. The significance of the correlations were evaluated by using a permutation test that compared them with null distributions of random correlations. For each biometric, the null distribution of correlations was created from 3,000 iterations where each iteration randomly shuffled the performance (response and reaction time) across the trials through the task. For a subject's threat detection performance to be counted as exhibiting significant correlation with a biometric, the correlations have to be significant for both threat types. If only for one threat type, the particular subject is counted as 0.5.

## 3. Results

**Figure 6:**
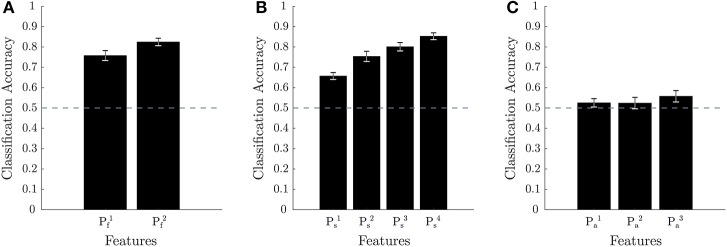
Cross-validation results for classification accuracy. 10-fold cross-validation results across 18 subjects with performance averaged across electrodes are shown in **(A,B)** for mental fatigue and stress, respectively. 3-fold cross-validation results across nine subjects with performance averaged across electrodes are shown in **(C)** for attention. The error bars for **(A–C)** are the standard error of the mean across subjects and the dotted line represents random chance accuracy.

Using *k*-fold cross-validation, the different feature sets were first evaluated to determine the best performing ones. Figure 6 provides the results of this approach for each of the three biometrics (namely, mental fatigue, stress, attention). Note that of the feature sets evaluated, $P_{f}^{1}$ for mental fatigue, $P_{s}^{1}$ and $P_{s}^{2}$ for stress, and $P_{a}^{1}$, $P_{a}^{2}$, and $P_{a}^{3}$ for attention have been previously proposed in the literature and are considered the current state-of-the-art. As the cross-validation accuracies for the various biometric models, as well as the biometric scores under various conditions, are not known to be other than normally distributed across the population, we employed a repeated measures ANOVA and t-tests to statistically analyze the various obtained results. Two-tailed paired sample t-tests showed that all the evaluated models for mental fatigue and stress performed significantly better than chance level of 50% ($P_{f}^{1}$: *p* < 1*e* − 8; $P_{f}^{2}$: *p* < 1*e* − 11; $P_{s}^{1}$: *p* < 1*e* − 8; $P_{s}^{2}$: *p* < 1*e* − 7; $P_{s}^{3}$: *p* < 1*e* − 10; $P_{s}^{4}$: *p* < 1*e* − 12). For the attention models, only $P_{a}^{3}$ performed better than chance at a trend level (*p* = 0.0739). Figure 6A shows that $P_{f}^{2}$ obtains the best performance for fatigue by utilizing EEG spectral power features from all the various physiological bands (namely, *delta* through *high-gamma*). A post-hoc two-tailed paired sample t-test comparing $P_{f}^{1}$ and $P_{f}^{2}$ was also very significant (*p* < 1*e* − 4). Figure 6B shows that $P_{s}^{4}$ improves over all prior feature sets by utilizing HRV spectral features together with the full range of EEG spectral power features. Post-hoc two-tailed paired sample t-tests comparing $P_{s}^{4}$ with each of the other stress models were very significant as well (with $P_{s}^{1}$: *p* < 1*e* − 7; with $P_{s}^{2}$: *p* < 1*e* − 3; with $P_{s}^{3}$: *p* < 1*e* − 3). Figure 6C shows $P_{a}^{3}$ performs slightly better than the other models for attention, but post-hoc two-tailed paired sample t-tests comparing the models were not significant when considering multiple comparisons ($P_{a}^{1}$ vs. $P_{a}^{3}$ : *p* = 0.0707; $P_{a}^{2}$ vs. $P_{a}^{3}$ : *p* = 0.0411).

In order to interpret the best GLM models for mental fatigue and stress (namely, $P_{f}^{2}$ and $P_{s}^{4}$), we looked at individual features at each channel for consistency across subjects (>50%) in being significant (*p* < 0.05) and the directionality of the correlation. As a result, we identified a subset of feature-channel combinations that related to the predicted mental states; see Table S15 for mental fatigue and Table S16 for stress. For mental fatigue, the most consistent feature for a positive influence is frontal *gamma*, and the most consistent feature for a negative influence is fronto-central *alpha*. For stress, the most consistent features for a positive influence are pre-frontal, central, and centro-parietal *theta*, and frontal *gamma*, and the most consistent features for a negative influence are fronto-central *delta*, frontal *beta*, and pre-frontal, fronto-central, central, and centro-parietal *alpha*. Note, however, that artifact removal is an imperfect process and there may still be EEG artifact remnants, especially those caused by slow eye movements (SEMs) and facial muscle activity. In particular, SEM artifacts are known to contaminate EEG spectral power in the *delta* band, and frontal electromyogram (EMG) artifacts are known to confound endogenous frontal *gamma* power.

**Figure 7:**
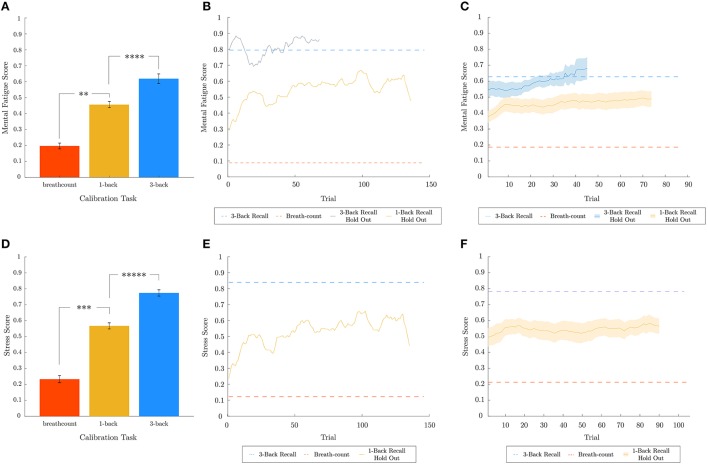
Regression analysis for mental fatigue and stress metrics. Leave-one-out cross-validated breath-count and 3-back results as well as 1-back hold out and 3-back hold out results are shown in **(A–C)** for mental fatigue and in **(D–F)** for stress, respectively. In **(A,D)**, two one-tailed paired sample *t*-tests are performed for mental fatigue and stress, respectively, to ensure 1-back task data is predicted to be intermediate between the bounds trained using the breath-count and 3-back task data. The corresponding *p*-values for mental fatigue are *p* < 1*e* − 4 (breath-count ≠ 1-back) and *p* < 0.005 (1-back ≠ 3-back), and for stress are *p* < 1*e* − 3 (breath-count ≠ 1-back) and *p* < 1*e* − 5 (1-back ≠ 3-back). **(B,E)** show the biometric predictions for a representative subject on the first half of 3-back recall task and the 1-back recall task for mental fatigue, and on the 1-back recall task for stress, respectively. The dashed red and blue lines in **(B,E)** correspond to the breath-count and 3-back scores from the respective models for the representative subject. Averaging the results from all subjects, **(C,F)** show the biometric predictions on held-out data across subjects. As in **(B,E)**, the dashed red and blues lines correspond to the breath-count and 3-back scores averaged across subjects. In **(C,F)**, the shaded regions depict standard error of the mean across subjects. ^**^p < 0.01, ^***^p < 0.001, ^****^p < 0.0001, ^*****^p < 0.00001.

In order to assess the predictive capabilities of the best biometric models for mental fatigue and stress within the continuous range between 0 and 1, a regression test was performed with data from the 1-back recall task, which was not used in training the models. The results for mental fatigue (top) and stress (bottom) are shown in Figure 7. The attention biometric was not evaluated similarly, due to the lack of data from a held-out task that would have corresponded with an intermediate attentional state. While the datasets for breath-count and 3-back recall tasks are utilized to train the mental fatigue and stress models for the lower and upper bounds, leave-one-out cross-validation was utilized to assess performance on the training data itself. In Figures 7A,D, the prediction scores averaged across subjects are shown for both training (breath-count and 3-back tasks) and test (1-back task) data. Two two-tailed paired sample t-tests were performed for metal fatigue and stress results to confirm that the intermediate (1-back) task predictions were between the upper and lower bounds. As *p*-values were less than 0.025 (correcting for two comparisons), we can conclude that the best biometric models for mental fatigue ($P_{f}^{2}$) and stress ($P_{s}^{4}$) predict an intermediate representation for the 1-back recall task. Figures 7B,E show the biometric predictions for a representative subject on the first half of 3-back recall task and the 1-back recall task for mental fatigue, and on the 1-back recall task for stress, respectively. And Figures 7C,F show the biometric predictions on held-out data across subjects. Model predictions on the 1-back recall task, averaged across subjects, for mental fatigue and stress were not correlated (*p* = 0.2645). For mental fatigue, as expected, there is a gradual increase through time in both 1-back and 3-back recall tasks. In contrast, stress does not change much over time with the level determined by the cognitive load of the task. Indeed, one-way repeated measures ANOVA on model predictions for the 1-back recall task revealed no effect of time for stress (*p* = 0.9273), but a very significant effect of time for mental fatigue (*p* < 1*e* − 7).

**Figure 8:**
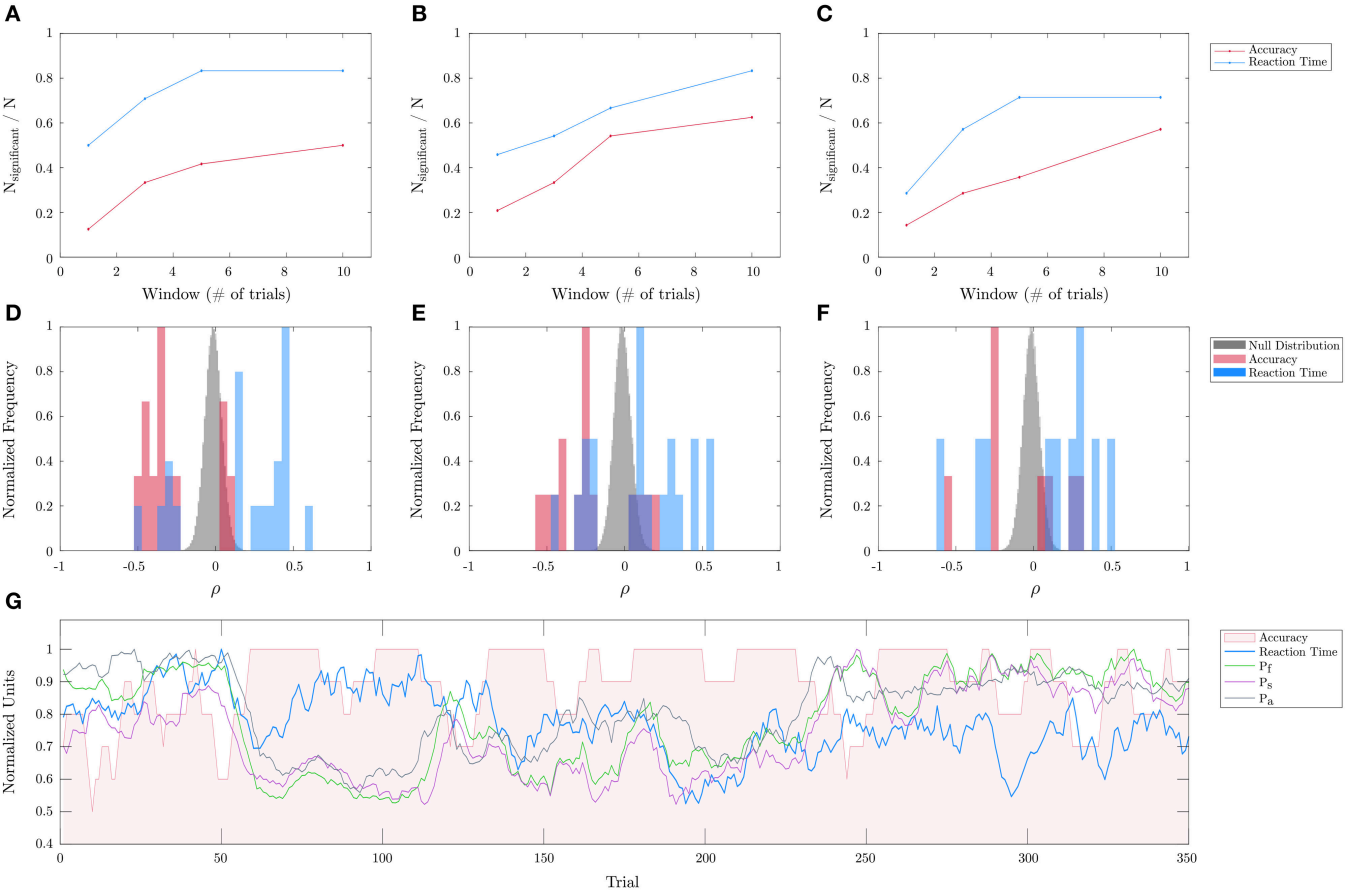
Threat detection task biometric evaluation. Evaluation of mental fatigue, stress, and attention for correlation with the temporal dynamics of performance on the threat detection task across different subjects (and over 23 experimental days) is shown. **(A–C)** shows the proportion of subjects exhibiting significant correlations for *P*_*f*_, *P*_*s*_, and *P*_*a*_, and for each performance metric (accuracy, reaction time) as a function of the integration window. **(D–F)** shows the distribution of the statistically significant accuracy (red) and response time (blue) correlation coefficients with respect to the overall null distributions (gray), all of which are normalized by their maximal frequencies for easier comparisons. **(G)** shows a 10-trial averaged prediction of each of the biometrics as well as the observed accuracies and reaction times across trials over 24 h for a representative subject.

In order to evaluate the efficacy of the personalized biometric models on a novel task, the best learned mental fatigue, stress, and attention models were applied to the threat detection task to correlate with performance fluctuations. The performance was assessed using different moving average windows in terms of number of trials (from 1 through 10). The corresponding biometric values are sampled and averaged from the temporal extents of the respective trials. As mentioned in the section 2.2, the significance of behavior-biometric correlations was determined using a permutation test. The top row of Figure 8 shows the proportion of subjects exhibiting significant (*p* < 0.05) correlations as a function of the moving average window for each of the three biometrics and for either performance metric (namely, accuracy and reaction time). It is shown there are a greater number of significant correlations for longer windows, and for reaction time compared to accuracy. Unlike accuracy, reaction time is a continuous metric allowing for less noisy estimates of performance with shorter windows. The second row depicts the distribution of significant correlation coefficients for each biometric (red: accuracy; blue: reaction time) against the overall null distribution (gray) across subjects and between the two performance metrics obtained from 3,000 iterations for the permutation test. Correlation data and permutation test assessment for each subject, biometric, window length, performance metric are provided in Tables S3–S14. Analyzing the significant correlation coefficients, it can be noted that they exhibit both positive and negative values away from the null distributions across subjects. This indicates that the biometrics have different effects on performance across subjects. The bottom row of Figure 8 illustrates the temporal dynamics of performance (accuracy, reaction time), averaged over 10 trials, as well as for each of the predicted biometrics for a representative subject across trials over 24 h in one of the two experiment days.

## 4. Discussion

The paper not only provides a unified pipeline for extracting a comprehensive mental state evaluation from a parsimonious set of sensors (only EEG and ECG), but also addresses the current limitations in assessing the efficacy of qualitative biometrics for individual subjects by proposing and employing a multi-level validation scheme for the biometric models by means of *k*-fold cross validation for discrete classification and regression testing for continuous prediction.

In this regard, we have demonstrated a new approach for classifying mental state using two calibration tasks (namely, breath-count and 3-back recall tasks) to construct biometric models that can be used to score and validate an individual's state in subsequent tasks. For both mental fatigue and stress, new feature sets have been identified that improve upon the performance of existing approaches. In particular, we see that using EEG spectral power features across the full range of physiological bands allows for better representation of all mental states with a committee of single-electrode evaluations. This is in contrast to existing approaches that either consider only a select few bands or have the many-to-one mapping problem when a proportion between frequency bands is used. For stress, the relatively worse-performing state-of-the-art $P_{s}^{1}$ model, which uses fine binning of the spectral bands, yields above-chance cross-validation accuracy, but because of relatively more number of input features likely requires more training examples to effectively learn a representation for the stress biometric. Nonetheless, our novel proposed $P_{s}^{4}$ model utilizing both EEG and HRV spectral power features in physiologically relevant bands provided the best cross-validation performance. Note that mental fatigue and stress models are built from essentially the same calibration task data, though the latter half of 3-back recall task is used to represent high mental fatigue and the whole of the 3-back recall task is used to represent high stress. This is based on the understanding that mental fatigue gradually increases through a tiring and cognitively demanding task (such as the 3-back), whereas stress becomes elevated on a faster time scale with increased cognitive load; see Cohen and Spacapan (1978) and Hancock (1989). Furthermore, HRV features extracted from ECG are only used for modeling stress and not mental fatigue. Future work should consider building a single label-agnostic biometric model that can predict task performance for individual users that envelopes the potentially idiosyncratic behavioral effects of traditional biometrics for the utility of closed-loop intervention applications. Also, the model inputs should consider all channels together and extract features that span multiple channels (e.g., coherence between channels in different frequency bands). And given that only a subset of features and channels seem to be related to the predicted mental states, further improvement in model performance can likely be obtained by focusing on the most promising features and channels; see Results section and Tables S15, S16.

Little can be said about the feature sets for the attention model due to small number of informative labels acquired from each subject. Across the nine subjects utilized for the attention analysis, few recorded greater than three mind-wandering events in the breath-count task, which resulted in the lack of sufficient data to train the models. The maximum classification accuracy achieved for any single subject was 65%. As such, the techniques proposed by Lum (1977), Feldman et al. (2010), and Braboszcz and Delorme (2011) require further improvement. The basic design of the breath-count task is good, but subject compliance might be improved with better instructions. For example, the facilitator might conduct a guided practice session beforehand, as few people have experience in concentrating on their breath. Additionally, subjects should not close their eyes during the task because they are more likely to fall asleep. And the importance of recognizing and recording mind-wandering should be stressed to the subjects. But it should be noted that these issues only affect the attention metric, since the stress and fatigue metrics reference the entire breath count data as the low level of each metric, and are independent of mind-wandering events.

Further, we employed a set of validation techniques to establish the efficacy of the biometrics in the absence of empirical data. We validated the component mental state metrics described here using three validation schemes: (1) Classical machine learning approach of *k*-fold cross-validation established the efficacy of discrete binary classification utilizing the biometric scoring with task-level labeling. (2) As confirmation of the validity of the continuous mental state scores, we evaluated the predictive performance of the best mental fatigue and stress models trained solely on the biometrics in a high state (3-back) and low state (breath-count). By holding out the intermediate state biometric data (1-back) during training, we could confirm that the biometrics provide reasonable values in novel conditions. Further, we demonstrated that when the models were run on holdout data for the breath-count, 1-back, and 3-back experiments, the regression scores fall into the appropriate ranges (relatively low, medium, or high, respectively). This demonstrates that the biometrics do provide a useful range and that the features utilized for training the models generalize beyond the discrete classification. (3) A permutation test against a novel task established that the proposed metrics are informative on a completely unrelated untrained behavioral task.

After training and validating the models on the calibration tasks, we demonstrated that the same models could be used to produce biometric assessments in a different real-world task (namely, the threat detection task, Figure 8). Beyond the obvious importance of the threat detection task to the military, it is of course important as well to law enforcement personnel (Sweet et al., 2017), baggage screeners (Basner et al., 2008), security guards (Cohen et al., 2008), operators of unmanned vehicles (Cummings et al., 2007), and others. As mentioned above, biometrics are only useful and task-relevant insofar as they are relatable to observable fluctuations in performance. In this context, it is important to be able to distinguish between task-related and non-task-related influences. For example, an office worker who observes a car wreck from his or her desk may be highly distracted yet show a high attention biometric, even though attention paid to the car wreck impairs performance on the relevant task of office work. However, for a driver who sees the car accident occurring on the road ahead, the resulting high level of attention is relevant. So the influence of a physiologically-determined attention metric on arbitrary task performance may be hard to discern. Examining the subjects that did not exhibit significant correlations between biometrics and performance on the novel threat detection task, we observe remaining artifacts in their neural and cardiac data that affect the decodability. Apart from differences in the amount of individual engagement in the task, there could also be large variations in neural and physiological responses between subjects as found by other researchers (Hockey et al., 2009; Ting et al., 2010). While the spread of the significant correlation coefficients was broad across subjects with seeming negative and positive modes, the presence of a statistically significant trend in one direction indicates that population-level biometric models could also be useful for an individual user lacking calibration data.

In conclusion, mental state monitoring is a critical component of current and future human-machine interfaces, including semi-autonomous driving and flying, air traffic control, decision aids, training systems, and will soon be integrated into ubiquitous products like cell phones and laptops. Based on the results presented in this paper, it is possible to integrate biometrics into augmented human-machine interfaces in a judicious way that can help to maximize task performance.

## Author contributions

AP, MH, PP, and SR performed the analyses, contributed to the interpretation of the results, and wrote the manuscript. AJ, NB, CR, and VC conducted the experiments and gave feedback on the manuscript.

### Conflict of interest statement

AP, MH, SR, and PP were employed by HRL Laboratories, LLC. SR, MH, and PP have a pending patent application on physiological assessment. The other authors declare that the research was conducted in the absence of any commercial or financial relationships that could be construed as a potential conflict of interest.
