# Supplementary material for: Mental State Assessment and Validation Using Personalized Physiological Biometrics
Source: Front Hum Neurosci. 2018 Jun 1;12:221. doi: 10.3389/fnhum.2018.00221 (PMC5992431; doi:10.3389/fnhum.2018.00221)
Supplement: Supplementary file 1 [file Table_1.PDF]

Supplementary Material: Mental state assessment and validation using personalized physiological biometrics

Aashish N. Patel [1], Michael D. Howard [1], Shane M. Roach [1], Aaron P. Jones [2], Natalie B. Bryant [2], Charles S. H. Robinson [2], Vincent P. Clark [2] and Praveen K. Pilly [1,*]

*Correspondence:
Dr. Praveen K. Pilly
pkpilly@hrl.com

1 SUPPLEMENTARY TABLES

.

S1 Table

Subject information on gender, age, and the availability of data for the various validation tests (k: k-fold cross-validation, r: regression testing, e: novel task evaluation).

Subject ID	1247	1364	2178	2335	3882	3901	4027	5281	5356
Gender	M	F	M	F	M	F	M	M	M
Age	23	21	22	18	24	19	19	19	21
Evaluation	k,r,e	k,r	k,r,e	k,e	k,r,e	k,r,e	k,e	k,r,e	k,r,e

Subject ID	5613	6174	6275	6425	7184	7843	8656	9513	9650
Gender	F	M	F	M	F	F	M	F	M
Age	22	19	19	19	20	19	39	19	22
Evaluation	k	k,r,e	k	k,e	k,r,e	k,r,e	k,r,e	k,r,e	k,r

S2 Table

Number of trials for each threat type that were available for evaluation of the biometric models on the threat detection task.

Subject ID	1247	2178	2335	3882	3901	4027	5281	5356	6174
Object Threat	221	210	150	150	210	180	210	150	210
Person Threat	150	210	225	240	210	150	270	150	150

Subject ID	6425	7184	7843	8656	9513
Object Threat	210	249	210	210	193
Person Threat	225	240	-	180	270

S3 Table

Results for Day 1 threat detection assessment for correlations between mental fatigue and reaction time.

		1247	2178	2335	3882	3901	4027	5281	5356
1-bin	p	0.000	0.002	0.031	0.754	0.160	0.050	0.040	0.000
	rho	0.459	0.151	-0.124	0.023	0.069	0.113	-0.104	0.232
	p-perm	0.000	0.002	0.013	0.379	0.085	0.025	0.017	0.000
3-bin	p	0.000	0.000	0.001	0.310	0.461	0.000	0.000	0.000
	rho	0.585	0.270	-0.185	-0.077	0.036	0.265	-0.208	0.348
	p-perm	0.000	0.000	0.000	0.152	0.227	0.000	0.000	0.000
5-bin	p	0.000	0.000	0.000	0.387	0.588	0.000	0.000	0.000
	rho	0.632	0.370	-0.236	-0.066	0.027	0.372	-0.281	0.413
	p-perm	0.000	0.000	0.000	0.190	0.304	0.000	0.000	0.000
10-bin	p	0.000	0.000	0.000	0.686	0.634	0.000	0.000	0.000
	rho	0.675	0.544	-0.298	-0.031	0.024	0.522	-0.441	0.486
	p-perm	0.000	0.000	0.000	0.341	0.326	0.000	0.000	0.000

		6174	6425	7184	7843	8656
1-bin	p	0.915	0.002	0.000	0.039	0.003
	rho	-0.006	0.180	0.226	0.119	0.156
	p-perm	0.453	0.002	0.000	0.017	0 .003
3-bin	p	0.063	0.000	0.000	0.000	0.000
	rho	-0.108	0.287	0.315	0.259	0.279
	p-perm	0.031	0.000	0.000	0.000	0.000
5-bin	p	0.010	0.000	0.000	0.000	0.000
	rho	-0.149	0.359	0.342	0.380	0.398
	p-perm	0.005	0.000	0.000	0.000	0.000
10-bin	p	0.000	0.000	0.000	0.000	0.000
	rho	-0.205	0.495	0.370	0.523	0.518
	p-perm	0.001	0.000	0.000	0.000	0.000

S4 Table

Results for Day 2 threat detection assessment for correlations between mental fatigue and reaction time.

		1247	2178	2335	3882	4027	5356	6174	6425	7184	8656	9513
1-bin	p	0.084	0.731	0.579	0.428	0.059	0.002	0.189	0.207	0.000	0.318	0.318
	rho	0.100	0.017	0.028	0.042	-0.100	0.177	-0.064	0.067	0.208	0.065	0.058
	p-perm	0.042	0.379	0.291	0.209	0.030	0.001	0.087	0.099	0.000	0.160	0.163
3-bin	p	0.043	0.088	0.031	0.909	0.000	0.000	0.038	0.013	0.000	0.996	0.276
	rho	0.117	0.084	0.109	0.006	-0.197	0.285	-0.102	0.131	0.321	0.000	0.063
	p-perm	0.026	0.043	0.013	0.442	0.000	0.000	0.025	0.007	0.000	0.493	0.140
5-bin	p	0.008	0.008	0.002	0.981	0.000	0.000	0.007	0.001	0.000	0.785	0.023
	rho	0.153	0.130	0.157	-0.001	-0.228	0.359	-0.131	0.179	0.358	-0.018	0.132
	p-perm	0.004	0.003	0.000	0.485	0.000	0.000	0.003	0.000	0.000	0.390	0.015
10-bin	p	0.000	0.000	0.000	0.455	0.000	0.000	0.001	0.000	0.000	0.201	0.000
	rho	0.216	0.210	0.243	-0.040	-0.205	0.505	-0.171	0.230	0.418	-0.084	0.304
	p-perm	0.000	0.000	0.000	0.221	0.000	0.000	0.000	0.000	0.000	0.098	0.000

S5 Table

Results for Day 1 threat detection assessment for correlations between stress and reaction time.

		1247	2178	2335	3882	3901	4027	5281	5356
1-bin	p	0.000	0.003	0.021	0.957	0.192	0.003	0.465	0.000
	rho	0.374	0.147	-0.133	-0.004	0.064	-0.171	-0.037	0.302
	p-perm	0.000	0.003	0.010	0.471	0.093	0.002	0.232	0.000
3-bin	p	0.000	0.000	0.004	0.133	0.004	0.000	0.141	0.000
	rho	0.506	0.258	-0.168	-0.113	0.141	-0.256	-0.075	0.457
	p-perm	0.000	0.000	0.001	0.068	0.001	0.000	0.073	0.000
5-bin	p	0.000	0.000	0.001	0.020	0.001	0.000	0.061	0.000
	rho	0.556	0.349	-0.189	-0.175	0.162	-0.315	-0.095	0.542
	p-perm	0.000	0.000	0.001	0.012	0.001	0.000	0.032	0.000
10-bin	p	0.000	0.000	0.000	0.011	0.000	0.000	0.007	0.000
	rho	0.607	0.508	-0.220	-0.195	0.189	-0.364	-0.137	0.629
	p-perm	0.000	0.000	0.000	0.004	0.000	0.000	0.004	0.000

		6174	6425	7184	7843	8656
1-bin	p	0.494	0.832	0.025	0.009	0.009
	rho	0.040	0.012	0.102	0.151	0.138
	p-perm	0.242	0.413	0.015	0.005	0.004
3-bin	p	0.642	0.232	0.000	0.000	0.000
	rho	0.027	-0.069	0.177	0.287	0.205
	p-perm	0.318	0.109	0.000	0.000	0.000
5-bin	p	0.171	0.090	0.000	0.000	0.000
	rho	0.080	-0.099	0.171	0.388	0.275
	p-perm	0.086	0.047	0.000	0.000	0.000
10-bin	p	0.009	0.054	0.000	0.000	0.000
	rho	0.153	-0.113	0.193	0.525	0.353
	p-perm	0.003	0.028	0.000	0.000	0.000

S6 Table

Results for Day 2 threat detection assessment for correlations between stress and reaction time.

		1247	2178	2335	3882	4027	5356	6174	6425	7184	8656	9513
1-bin	p	0.060	0.040	0.977	0.947	0.531	0.017	0.837	0.072	0.022	0.090	0.404
	rho	0.109	0.100	0.001	0.004	0.033	0.138	-0.010	0.095	0.111	0.110	0.048
	p-perm	0.030	0.017	0.474	0.477	0.279	0.010	0.395	0.032	0.014	0.043	0.197
3-bin	p	0.023	0.000	0.140	0.352	0.975	0.000	0.402	0.057	0.000	0.323	0.612
	rho	0.132	0.182	0.075	-0.049	0.002	0.242	-0.041	0.101	0.222	0.064	0.030
	p-perm	0.014	0.000	0.070	0.176	0.499	0.000	0.209	0.026	0.000	0.167	0.325
5-bin	p	0.003	0.000	0.036	0.340	0.102	0.000	0.127	0.040	0.000	0.597	0.893
	rho	0.171	0.240	0.106	-0.051	-0.087	0.313	-0.075	0.109	0.278	0.035	0.008
	p-perm	0.002	0.000	0.021	0.164	0.049	0.000	0.061	0.015	0.000	0.285	0.468
10-bin	p	0.000	0.000	0.003	0.115	0.015	0.000	0.002	0.014	0.000	0.657	0.283
	rho	0.241	0.325	0.152	-0.084	-0.129	0.428	-0.151	0.131	0.373	-0.029	-0.063
	p-perm	0.000	0.000	0.002	0.057	0.008	0.000	0.001	0.008	0.000	0.320	0.147

S7 Table

Results for Day 1 threat detection assessment for correlations between attention and reaction time.

		2335	3882	3901	4027	5281	6174	6425	7184
1-bin	p	0.578	0.232	0.397	0.059	0.958	0.351	0.000	0.259
	rho	0.032	0.090	-0.041	0.109	-0.003	0.054	-0.217	0.052
	p-perm	0.291	0.107	0.201	0.028	0.465	0.174	0.000	0.126
3-bin	p	0.444	0.212	0.021	0.000	0.013	0.025	0.000	0.487
	rho	0.045	0.094	-0.113	0.313	0.125	0.130	-0.313	0.032
	p-perm	0.222	0.105	0.010	0.000	0.008	0.011	0.000	0.242
5-bin	p	0.536	0.020	0.000	0.000	0.000	0.004	0.000	0.711
	rho	0.036	0.175	-0.189	0.418	0.239	0.167	-0.383	0.017
	p-perm	0.270	0.009	0.000	0.000	0.000	0.002	0.000	0.344
10-bin	p	0.603	0.000	0.000	0.000	0.000	0.000	0.000	0.925
	rho	0.031	0.390	-0.249	0.578	0.390	0.224	-0.513	-0.004
	p-perm	0.294	0.000	0.000	0.000	0.000	0.000	0.000	0.461

S8 Table

Results for Day 2 threat detection assessment for correlations between attention and reaction time.

		2335	3882	4027	6174	6425	7184
1-bin	p	0.001	0.422	0.225	0.592	0.000	0.039
	rho	0.163	-0.042	-0.064	0.026	-0.275	0.101
	p-perm	0.001	0.209	0.096	0.274	0.000	0.020
3-bin	p	0.000	0.957	0.463	0.058	0.000	0.000
	rho	0.279	-0.003	-0.039	0.093	-0.293	0.176
	p-perm	0.000	0.492	0.228	0.032	0.000	0.000
5-bin	p	0.000	0.807	0.898	0.010	0.000	0.000
	rho	0.365	-0.013	-0.007	0.126	-0.266	0.266
	p-perm	0.000	0.392	0.448	0.006	0.000	0.000
10-bin	p	0.000	0.698	0.899	0.001	0.000	0.000
	rho	0.486	-0.021	0.007	0.171	-0.286	0.334
	p-perm	0.000	0.353	0.451	0.000	0.000	0.000

S9 Table

Results for Day 1 threat detection assessment for correlations between mental fatigue and accuracy.

		1247	2178	2335	3882	3901	4027	5281	5356
1-bin	p	0.000	0.053	0.423	0.670	0.072	0.720	0.509	0.029
	rho	-0.177	-0.095	0.046	-0.032	-0.088	0.021	-0.033	-0.126
	p-perm	0.000	0.026	0.215	0.318	0.032	0.361	0.262	0.014
3-bin	p	0.000	0.000	0.367	0.450	0.498	0.427	0.051	0.000
	rho	-0.256	-0.182	0.052	-0.057	0.033	0.046	-0.099	-0.219
	p-perm	0.000	0.000	0.184	0.228	0.245	0.216	0.028	0.001
5-bin	p	0.000	0.000	0.387	0.104	0.046	0.382	0.006	0.000
	rho	-0.298	-0.248	0.050	-0.123	0.098	0.051	-0.139	-0.255
	p-perm	0.000	0.000	0.199	0.057	0.022	0.190	0.002	0.000
10-bin	p	0.000	0.000	0.511	0.011	0.000	0.116	0.000	0.000
	rho	-0.360	-0.315	0.039	-0.193	0.190	0.092	-0.286	-0.377
	p-perm	0.000	0.000	0.262	0.007	0.000	0.053	0.000	0.000

		6174	6425	7184	7843	8656
1-bin	p	0.676	0.079	0.685	0.703	0.812
	rho	0.024	-0.102	-0.019	0.022	-0.013
	p-perm	0.327	0.042	0.350	0.346	0.407
3-bin	p	0.980	0.001	0.439	0.625	0.878
	rho	0.001	-0.192	-0.035	-0.028	0.008
	p-perm	0.484	0.000	0.223	0.295	0.457
5-bin	p	0.643	0.000	0.905	0.217	0.872
	rho	0.027	-0.241	0.005	-0.072	0.009
	p-perm	0.316	0.000	0.441	0.114	0.440
10-bin	p	0.024	0.000	0.016	0.070	0.642
	rho	0.133	-0.281	0.111	-0.106	0.025
	p-perm	0.016	0.000	0.012	0.030	0.309

S10 Table

Results for Day 2 threat detection assessment for correlations between mental fatigue and accuracy.

		1247	2178	2335	3882	4027	5356	6174	6425	7184	8656	9513
1-bin	p	0.724	0.742	0.889	0.004	0.426	0.177	0.925	0.389	0.367	0.822	0.084
	rho	-0.021	-0.016	-0.007	-0.149	0.042	-0.078	0.005	0.046	-0.044	0.015	-0.100
	p-perm	0.365	0.378	0.442	0.002	0.225	0.091	0.451	0.191	0.177	0.410	0.053
3-bin	p	0.857	0.041	0.800	0.000	0.017	0.002	0.726	0.939	0.199	0.824	0.069
	rho	-0.011	-0.100	-0.013	-0.216	0.126	-0.181	0.017	0.004	-0.063	-0.015	-0.105
	p-perm	0.441	0.024	0.398	0.000	0.007	0.001	0.340	0.471	0.102	0.405	0.031
5-bin	p	0.751	0.002	0.790	0.000	0.014	0.000	0.613	0.702	0.357	0.784	0.147
	rho	-0.019	-0.149	-0.013	-0.310	0.130	-0.236	0.025	-0.020	-0.045	-0.018	-0.084
	p-perm	0.365	0.001	0.379	0.000	0.007	0.000	0.304	0.344	0.185	0.394	0.074
10-bin	p	0.419	0.000	0.632	0.000	0.117	0.000	0.615	0.616	0.996	0.969	0.091
	rho	-0.048	-0.238	0.024	-0.440	0.084	-0.281	0.025	-0.027	0.000	-0.003	-0.099
	p-perm	0.217	0.000	0.314	0.000	0.055	0.000	0.311	0.309	0.490	0.478	0.046

S11 Table

Results for Day 1 threat detection assessment for correlations between stress and accuracy.

		1247	2178	2335	3882	3901	4027	5281	5356
1-bin	p	0.004	0.015	0.170	0.315	0.376	0.149	0.346	0.005
	rho	-0.137	-0.118	0.079	-0.075	-0.043	0.084	-0.048	-0.163
	p-perm	0.003	0.006	0.087	0.150	0.186	0.080	0.173	0.002
3-bin	p	0.000	0.000	0.012	0.195	0.451	0.157	0.057	0.000
	rho	-0.205	-0.237	0.145	-0.098	-0.037	0.082	-0.096	-0.278
	p-perm	0.000	0.000	0.006	0.106	0.229	0.076	0.029	0.000
5-bin	p	0.000	0.000	0.001	0.080	0.681	0.042	0.041	0.000
	rho	-0.244	-0.322	0.199	-0.132	-0.020	0.118	-0.104	-0.348
	p-perm	0.000	0.000	0.000	0.042	0.351	0.020	0.015	0.000
10-bin	p	0.000	0.000	0.000	0.033	0.745	0.002	0.004	0.000
	rho	-0.302	-0.416	0.299	-0.163	-0.016	0.185	-0.146	-0.488
	p-perm	0.000	0.000	0.000	0.022	0.380	0.001	0.002	0.000

		6174	6425	7184	7843	8656
1-bin	p	0.529	0.031	0.911	0.805	0.622
	rho	-0.037	-0.125	0.005	-0.014	0.026
	p-perm	0.279	0.019	0.470	0.403	0.320
3-bin	p	0.073	0.000	0.929	0.161	0.213
	rho	-0.104	-0.206	0.004	-0.081	0.066
	p-perm	0.030	0.000	0.483	0.071	0.098
5-bin	p	0.020	0.000	0.134	0.033	0.139
	rho	-0.135	-0.207	0.069	-0.124	0.079
	p-perm	0.010	0.000	0.069	0.019	0.068
10-bin	p	0.000	0.007	0.000	0.003	0.167
	rho	-0.240	-0.157	0.222	-0.173	0.074
	p-perm	0.000	0.005	0.000	0.002	0.077

S12 Table

Results for Day 2 threat detection assessment for correlations between stress and accuracy.

		1247	2178	2335	3882	4027	5356	6174	6425	7184	8656	9513
1-bin	p	0.711	0.089	0.824	0.043	0.089	0.564	0.835	0.959	0.954	0.173	0.360
	rho	-0.021	-0.083	-0.011	-0.107	-0.090	-0.033	-0.010	0.003	-0.003	-0.088	-0.053
	p-perm	0.361	0.050	0.401	0.018	0.036	0.273	0.484	0.467	0.455	0.083	0.129
3-bin	p	0.901	0.000	0.759	0.004	0.167	0.064	0.735	0.546	0.377	0.032	0.888
	rho	-0.007	-0.181	-0.016	-0.153	-0.073	-0.108	0.017	0.032	-0.043	-0.139	0.008
	p-perm	0.466	0.000	0.379	0.001	0.081	0.037	0.344	0.270	0.188	0.014	0.423
5-bin	p	0.741	0.000	0.764	0.000	0.247	0.021	0.553	0.634	0.463	0.008	0.313
	rho	-0.019	-0.230	-0.015	-0.249	-0.061	-0.134	0.029	0.025	-0.036	-0.171	0.059
	p-perm	0.365	0.000	0.373	0.000	0.120	0.011	0.274	0.330	0.232	0.005	0.152
10-bin	p	0.352	0.000	0.834	0.000	0.813	0.153	0.954	0.771	0.721	0.007	0.011
	rho	-0.055	-0.332	0.011	-0.364	0.013	-0.084	-0.003	-0.016	-0.018	-0.176	0.148
	p-perm	0.184	0.000	0.414	0.000	0.398	0.085	0.476	0.379	0.358	0.003	0.006

S13 Table

Results for Day 1 threat detection assessment for correlations between attention and accuracy.

		2335	3882	3901	4027	5281	6174	6425	7184
1-bin	p	0.261	0.217	0.653	0.053	0.177	0.565	0.006	0.337
	rho	0.065	-0.092	0.022	-0.112	0.068	-0.033	0.159	-0.044
	p-perm	0.130	0.098	0.338	0.028	0.084	0.284	0.003	0.161
3-bin	p	0.457	0.216	0.005	0.006	0.099	0.417	0.000	0.274
	rho	0.043	-0.093	0.137	-0.160	0.084	-0.047	0.249	-0.050
	p-perm	0.230	0.106	0.002	0.004	0.051	0.203	0.000	0.127
5-bin	p	0.492	0.100	0.000	0.001	0.018	0.179	0.000	0.219
	rho	0.040	-0.124	0.229	-0.184	0.120	-0.078	0.304	-0.056
	p-perm	0.252	0.048	0.000	0.000	0.009	0.100	0.000	0.108
10-bin	p	0.580	0.132	0.000	0.004	0.000	0.003	0.000	0.301
	rho	0.033	-0.116	0.352	-0.168	0.189	-0.174	0.348	-0.048
	p-perm	0.284	0.067	0.000	0.002	0.000	0.002	0.000	0.142

S14 Table

Results for Day 2 threat detection assessment for correlations between attention and accuracy.

		2335	3882	4027	6174	6425	7184
1-bin	p	0.522	0.049	0.572	0.294	0.086	0.915
	rho	-0.032	-0.104	0.030	-0.051	0.090	-0.005
	p-perm	0.248	0.027	0.287	0.149	0.045	0.445
3-bin	p	0.946	0.000	0.550	0.676	0.185	0.806
	rho	-0.003	-0.220	0.032	-0.021	0.070	0.012
	p-perm	0.483	0.000	0.270	0.333	0.099	0.399
5-bin	p	0.821	0.000	0.654	0.832	0.430	0.425
	rho	-0.012	-0.302	-0.024	-0.010	0.042	0.039
	p-perm	0.409	0.000	0.314	0.419	0.207	0.205
10-bin	p	0.931	0.000	0.003	0.664	0.008	0.071
	rho	0.004	-0.454	-0.156	-0.022	0.140	0.089
	p-perm	0.469	0.000	0.001	0.330	0.005	0.038

S15 Table

Coefficients from the best GLM model for mental fatigue (namely, P_f^2) for features that show the most consistency across subjects ($> 50\%$) in being significant ($p < 0.05$) and the directionality of the correlation. For α at FC6 and FC1, one subject with outlier coefficients was excluded for assessing mean and standard deviation.

Feature	Channel	Consistency (%)	Mean Coefficient	Std. Coefficient
γ	F4	55.56	27.52	17.81
α	FC6	55.56	-11.01	7.68
α	FC1	55.56	-10.5	8.03

S16 Table

Coefficients from the best GLM model for stress (namely, P_s^4) for features that show the most consistency across subjects ($> 50\%$) in being significant ($p < 0.05$) and the directionality of the correlation. For γ and β at Fz, one subject with outlier coefficients was excluded for assessing mean and standard deviation.

Feature	Channel	Consistency (%)	Mean Coefficient	Std. Coefficient
θ	Fp2	55.56	1.50	0.61
θ	Cz	55.56	1.82	0.81
θ	CP5	55.56	1.46	0.80
γ	Fz	55.56	18.6	12.02
δ	FC6	55.56	-0.41	0.20
α	CP6	55.56	-2.44	2.81
α	C4	61.11	-2.27	2.08
α	Fp2	66.67	-3.41	1.96
α	Fp1	66.67	-2.96	1.59
α	CP2	55.56	-2.25	1.31
α	FC5	55.56	-2.44	2.09
β	Fz	55.56	-8.24	2.66
